# Effect of hemoglobin content on cerebral oxygen saturation during surgery for scoliosis in pediatric patients

**DOI:** 10.1186/s12871-021-01382-x

**Published:** 2021-06-01

**Authors:** Lin Liu, Zhipeng Qiang, Jianmin Zhang, Yi Ren, Xin Zhao, Wenya Fu, Zhong Xin, Zenghua Xu, Fang Wang, Lijing Li, Nan Zou, Xuemei Zhang, Lei Feng, Shuxuan Ma

**Affiliations:** 1 Department of Anesthesiology, Beijing Children’s Hospital, Capital Medical University, National Center for Children’s Health, Beijing, China; 2 Department of Anesthesiology, Jining NO.1 People’s Hospital, Jining Medical University, Jining, Shandong China; 3 Department of Orthopedics, Beijing Children’s Hospital, Capital Medical University, National Center for Children’s Health, Beijing, China; 4 Department of Transfusion Medicine, Beijing Children’s Hospital, Capital Medical University, National Center for Children’s Health, Beijing, China

**Keywords:** Pediatric patients, Regional cerebral oxygen saturation, Hemoglobin

## Abstract

**Background:**

Although regional cerebral oxygen saturation (rScO_2_) monitoring has been widely used in clinical practice, the relationship between hemoglobin (dHB) content and rScO_2_ is incompletely understood. The aim of this study was to analyze the effect of hemoglobin content on rScO_2_ in pediatric patients undergoing general anesthesia for correction of scoliosis.

**Methods:**

Ninety-two pediatric patients aged 3 to 14 years undergoing scoliosis correction surgery were enrolled. Continuous monitoring of bilateral regional cerebral oxygen saturation by near-infrared spectroscopy (NIRS, CASMED, USA) was performed after entering the operation room. rScO_2_ was recorded when the patients entered the operating room (T_0_, baseline), after anesthesia induced intubation (T_1_), and after radial artery puncture (T_2_). The lowest value of rScO_2_ during surgery was also recorded. The arterial blood pressure (ABP), heart rate (HR), pulse oxygen saturation (SpO_2_), end tidal carbon dioxide partial pressure (PetCO_2_) were continuously recorded. Patients were classified as low rScO_2_ or high rScO_2_ group according to whether the lowest intraoperative rScO_2_ was 15% lower than the baseline value. An analysis and comparison of differences in hemoglobin content in these two groups was carried out.

**Results:**

The preoperative hemoglobin-postoperative hemoglobin of patients in the high rScO_2_ group was significantly lower than that in the low rScO_2_ group (t = − 7.86, *p* < 0.01), the amount of bleeding during the operation was also less than that in the low rScO_2_ group (t = − 6.05, *p* < 0.01), and the systolic pressure of patients was higher than that in the low rScO_2_ group (t = 4.27, *p* < 0.01).

**Conclusions:**

The decrease in hemoglobin level which occurs during surgery leads to a decrease in cerebral oxygen saturation. In order to ensure patient safety during surgery, it is necessary to carry out volume management and appropriate transfusion and fluid replacement in a timely manner.

**Trial registration:**

Chinese Clinical Trial Registry, ChiCTR1800016359. Registered 28 May 2018.

## Background

Near-infrared spectroscopy (NIRS) is a noninvasive technology, which was first proposed by Jobsis in 1997 [1]. NIRS has been widely used to monitor regional cerebral oxygen saturation (rScO_2_), and NIRS may reflect the balance between cerebral oxygen demand and oxygen supply [2, 3]. During surgery for correction of scoliosis, there is typically an increase in bleeding when the osteotomy is carried out and pedicle screws are inserted. At this time, hemoglobin may decrease significantly and hemodynamics may become unstable, and this results in high risk for cerebral desaturation due to decreased perfusion of brain tissue. Some studies have shown that cerebral hypoperfusion in infants during the perioperative period is closely related to the occurrence of postoperative neurologic complications and prolonged hospital stays [4].

At this time, most research focuses on cerebral oxygen saturation in cardiac and vascular surgery. There have been few studies on the relationship of intraoperative hemorrhage, decrease in hemoglobin and changes in cerebral oxygen saturation in correction of scoliosis in pediatric patients.

The aim of this study was to demonstrate whether the decrease in hemoglobin during surgery for correction of scoliosis affects cerebral oxygen saturation in children. This has potential to provide a reference for intraoperative blood transfusion and fluid therapy.

## Methods

A total of 92 patients undergoing posterior spinal fusion for idiopathic scoliosis under general anesthesia between March 2019 and July 2019 were enrolled in this study. These patients were 3 to 14 years old, and had American Society of Anesthesiologists physical status I to III. All surgeries lasted for more than 2 h. Patients with a history of cervical spine disease, congenital carotid artery disease (congenital stenosis or malformation), congenital heart disease, or a scoliosis segment above the third thoracic vertebra were excluded. Patients were divided into high regional cerebral oxygen saturation (H-rScO_2_) and low cerebral oxygen saturation (L-rScO_2_) groups. According to whether the decrease in minimum rScO_2_ was more than 15% below the baseline. H-rScO_2_ is defined as the decrease in rScO2min is no more than 15% lower than the baseline or the value of rScO_2_min is 15% lower than the baseline but the duration is less than 2 min. L-rScO_2_ is defined as the value of rScO_2_min is lower than the basic value by more than 15% and the duration is no less than 2 min.

Using the FORE-SIGHT® MC-2030C NIRS monitor (NIRS, CASMED, USA) with the medium sensor (source-detector separation 12 mm and 40 mm) were placed on the forehead bilaterally and adjusted as necessary to obtain consistent readings. The sensor of the Fore-sight monitor is attached to the child’s bilateral eyebrow arch 1 ~ 2 cm, and continuously monitors the left and right sides of rScO_2_. The bilateral cerebral oxygen saturation was monitored continuously when the patient entered the operation room. All patients were subjected to heart rate (HR), noninvasive blood pressure (NBP), pulse oxygen saturation (SpO_2_) and bispectral index (BIS). After induction of anesthesia, arterial blood pressure (ABP) and end tidal carbon dioxide partial pressure (PetCO_2_) were also continuously monitored.

Anesthesia was induced by atropine (0.01 mg/kg), propofol (3 mg/kg), sufentanil (0.5 μg/kg), and rocuronamine (0.6 mg/kg), and was maintained with propofol (8–10 mg/kg/h) and remifentanil (0.2–0.4 μg/kg/min). After intubation the tidal volume was adjusted to 8–10 mL/kg and the respiratory rate was adjusted to maintain PetCO_2_ at 35–45 mmHg. During the operation, the inhaled oxygen concentration was adjusted to 70%, SpO_2_ was maintained above 95%, and the BIS value was controlled to within 40–60 to ensure an appropriate depth of anesthesia. During the operation, propofol was used to control the depth of anesthesia and remifentanil was used to control patient blood pressure. ABP was maintained within 15% of the baseline level. When blood pressure drops, norepinephrine infusion can be pumped at 0.1 μg/kg/min at the beginning, and then reduced to 0.01 μg/kg/min. Atropine 0.01–0.02 mg/kg was given if the heart rate decreased by more than 15%. One hour after entering the operation room, a 20 mL/kg injection of compound sodium lactate and sorbitol was used to give rapid expansion of blood volume. In cases of low blood volume, hydroxyethyl starch was used to expand the volume by 5–10 mL/kg. A cell saver machine was used for blood recovery. Decisions as to whether to infuse concentrated red blood cells and fresh frozen plasma were made based on the patient hemoglobin level. Intraoperative red blood cell volume (Hct) was maintained above 25%. When Hct was lower than 25%, transfusion of autologous blood started after washing. After that, allogeneic red blood cells are supplemented, and plasma is transfused at a ratio of 1:1, if Hct was still lower than 25%. A further determination as to whether the concentrated red blood cells required supplementation was made according to the results of blood gas analysis, rScO_2_ was recorded when the patient entered the operating room (T_0_, baseline), after anesthesia induced intubation (T_1_), after radial artery puncture (T_2_), and the lowest value of rScO_2_ during the operation was also recorded. Systolic blood pressure (SBP), HR, SpO_2_ and PetCO_2_ values were recorded continuously. Hemoglobin content was recorded before the surgery, the hemoglobin value at the time of the lo west rScO_2_ and after the operation. Quantities of bleeding and transfusion fluid were recorded.

### Statistical analysis

The main outcome index was set as the difference in hemoglobin level before and after surgery. The results of pre-experiment obtained as the mean values for rScO_2_ in the L-ScO_2_ and H-ScO_2_ groups were 3.50 ± 1.19 and 2.63 ± 1.23, respectively. The sample size was calculated based on comparison of mean value between two independent groups. I addition, we set the type I error α of the two-tailed test as 0.05, type II error β as 0.1, and the ratio of observers between L-ScO_2_ and H-ScO_2_ as 1:1.5. The sample size calculation formula is as presented below:

$$ n1=\frac{k+1}{k}{\left[\frac{{\left({t}_{\alpha }+{t}_{\beta}\right)}^2s}{\delta}\right]}^2\kern0.5em n2=k\ast n1 $$

*n1 is* L-ScO_2_group, *n2* is H-ScO_2_ group, *k* is 1.5, *s* is the combined standard deviation of the two groups, and *δ* is the difference between the mean value of the two groups. The calculated sample size of the L-ScO_2_ and H-ScO_2_ groups were 35 and 53. Taking the missing data into consideration, the sample size was increased by 10%, and the sample size is n1 = 39 persons, n2 = 59 persons. Because of the data loss during the experiment, the final sample size of the L-ScO_2_ and H-ScO_2_ groups were 35 and 57. The statistical calculations were done using the Statistical Package for the Social Sciences (SPSS) version 24.0. Data are expressed as mean ± standard deviation for normally distributed continuous variables. Continuous variables that did not conform to the normal distribution were represented by the median and quartile. Comparison of cerebral oxygen saturation on the left and right sides was carried out with the paired *t*-test. The significance of differences between two groups was determined by the *t*-test for independent samples. Multiple regression analysis was used to determine the risk factors for cerebral oxygen saturation reduction. The relationship between age, body mass index (BMI) and cerebral oxygen saturation was assessed using the Spearman correlation analysis. *p* < 0.05 was considered statistically significant.

## Results

### Patient characteristics

Ninety-two patients were evaluated. Table 1 shows a comparison of general data for the H-rScO_2_ and L-rScO_2_ groups. There was no significant difference in general data between the two groups.

**Table 1:** Patient characteristics [^−^x ± s]

	L-rScO_2_ group (*n* = 35)	H-rScO_2_ group (*n* = 57)
**Sex (M/F)**	19/16	28/29
**Age (years)**	9.43 ± 4.12	8.53 ± 3.34
**Height (cm)**	128.54 ± 24.63	124.10 ± 23.66
**Body weight (kg)**	30.27 ± 14.13	30.85 ± 12.89
**Pre-op HB(g/L)**	12.21 ± 1.35	12.17 ± 1.08
**B-left rScO**_**2**_	80.6 ± 3.32	79.54 ± 3.90
**B-right rScO**_**2**_	81.29 ± 3.49	80.11 ± 4.04

*H-rScO*_*2*_ High regional cerebral oxygen saturation, *L-rScO*_*2*_ Low regional cerebral oxygen saturation, *Pre-op HB* Preoperative hemoglobin value, *B-left rScO*_*2*_ Baseline left regional cerebral oxygen saturation_**,**_ *B-right rScO*_*2*_ Baseline right regional cerebral oxygen saturation.

### Perioperative characteristics

As basic hemoglobin content differs in patients of different ages, the correlation between hemoglobin and cerebral oxygen saturation is expressed by the dHB (preoperative hemoglobin-postoperative hemoglobin).

The dHB of the patients in the low cerebral oxygen saturation group was significantly higher than that in the high cerebral oxygen saturation group (t = − 7.86, *p* < 0.01), the amount of bleeding during the operation was also higher than that in the high rScO_2_ group (t = − 6.05, *p* < 0.01). The SBP of patients in the high cerebral oxygen saturation group was significantly higher than that of patients in the low cerebral oxygen saturation group (t = 4.27, *p* < 0.01). There was no significant difference in HR, SpO_2_ or PetCO_2_ between these two groups (*p* > 0.05) (Table 2).

**Table 2:** Perioperative characteristics [^−^x ± s]

	L-rScO_2_ group (*n* = 35)	H-rScO_2_ group (*n* = 57)
**Fluid infusion volume (mL)**	1825.89 ± 781.65	1435.00 ± 671.90
**Hemorrhagia amount (mL)**	1084.86 ± 584.05	504.58 ± 336.82
**dHB (g/L)**	3.50 ± 1.23	1.57 ± 1.10
**SBP (mmHg)**	79.31 ± 11.16	89.35 ± 10.83
**HR (beats/min)**	94.54 ± 14.96	92.79 ± 12.50
**SpO**_**2**_	99.94 ± 0.23	99.79 ± 0.75
**PetCO**_**2**_ **(mmHg)**	36.82 ± 3.57	37.29 ± 2.76

*HrScO*_*2*_ High regional cerebral oxygen saturation, *L-rScO*_*2*_ Low regional cerebral oxygen saturation, *SBP* Systolic blood pressure, *HR* Heart rate, *SpO*_*2*_ Pulse oxygen saturation, *PetCO*_*2*_ End-expiratory carbon dioxide, *dHB* Preoperative hemoglobin-postoperative hemoglobin.

**Fig. 1:**
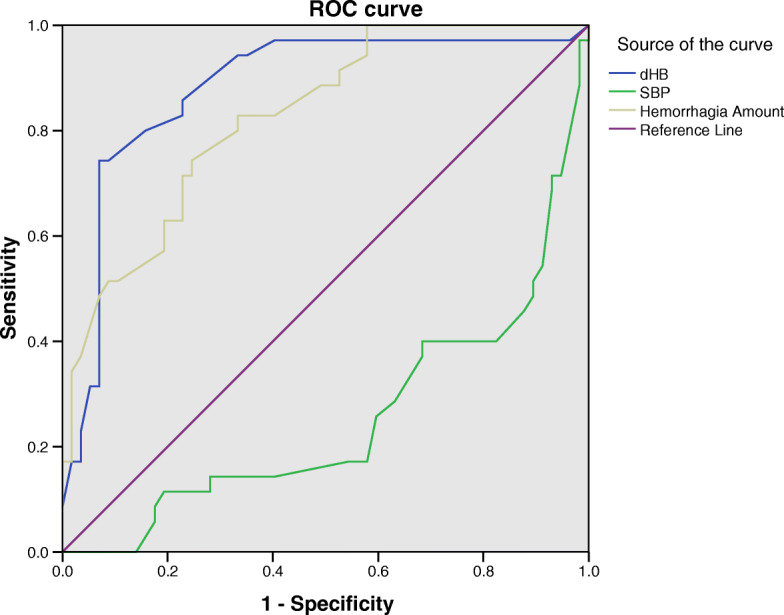
ROC curve

According to the logistic regression analysis, SBP reduction, intraoperative hemoglobin loss and intraoperative hemorrhage are the risk factors related to the reduction of patient rScO_2_. The OR value of dHB indicates that when increasing amounts of hemoglobin are lost cerebral oxygen saturation reduction are at increases risk (Table 3). The area under the ROC curve (AUC) was obtained as 0.88 and 95% confidence interval (CI) ranges from 1.55 to 5.04 (Fig. 1).

**Table 3:** Logistic regression analysis

	Β value	Standard error	***p*** value	OR value (95% CI)	AUC Area under curve
SBP	−0.10	0.03	0.00	0.91 (0.85–0.97)	0.25
dHB	1.03	0.30	0.00	2.79 (1.55–5.04)	0.88
Hemorrhagia amount	0.03	0.00	0.00	1.00 (1.00–1.01)	0.82

*SBP* Systolic blood pressure, *dHB* Preoperative hemoglobin-postoperative hemoglobin, *OR* Odds ratio, *CI* Confidence interval, ROC curve, *AUC* Area under curve.

## Discussion

The surgical correction of idiopathic scoliosis has particular problems. Osteotomy and hemivertebra resection typically cause massive hemorrhage. This leads to a decrease in hemoglobin which affects cerebral perfusion, may leads to hypoxia of brain tissue and postoperative neurologic complications. A previous study found that if brain metabolism is constant, cerebral oxygen saturation directly reflects cerebral blood flow [5]. Cerebral oxygen saturation monitoring has therefore been widely used in adult and pediatric surgery [6, 7]. At present, there is no absolute reference value for cerebral oxygen saturation, but it has been reported that the risk of brain injury is significantly increased when the cerebral oxygen saturation drops below 50% of the baseline [8, 9], and reduction greater than 20% from the baseline value during surgery is associated with postoperative neurologic changes at 1 year of age [10]. Impaired cerebral oxygen supply and demand balance during surgery may lead to neurological complications [11].

To eliminate the confounding effects of anesthetics and the concentration of inhaled oxygen in evaluating the results of this study, cerebral oxygen saturation was taken into consideration after radial artery puncture, rather than using the preoxygenation value as the reference for investigation of the effects of hemoglobin concentration.

Our study shows that reduction of hemoglobin during surgery is a risk factor for reduction of cerebral oxygen saturation. This is related to the characteristics of hemoglobin. The combination of hemoglobin and oxygen reflects the oxygen carrying capacity of red blood cells [12]. Surgery results in a decrease of hemoglobin content, and combined with concurrent large volume fluid supplementation leads to hemodilution and to decreases in the hemoglobin concentration. These factors together lead to a decrease in oxygen carrying capacity. This may reduce the oxygen supply to the brain tissue and decrease cerebral oxygen saturation.

We also found that SBP and cerebral oxygen saturation show a statistically significance relationship. When the rScO_2_ value was lowest, the SBP of the H-rScO_2_ group was significantly higher than that of the L-rScO_2_ group, and the binary Logistic regression analysis showed that there was statistical significance between the decrease of the SBP and the decrease of the cerebral oxygen saturation. Reduction in rScO_2_ values was accompanied by reduction of SBP. This shows that cerebral blood vessels have a mechanism for self-regulation. Cerebral perfusion pressure equals mean arterial pressure-intracranial pressure. When mean arterial pressure is maintained in the range of 55–65 mmHg, a small range fluctuation of blood pressure does not significantly affect cerebral perfusion. However, in the course of this study, insertion of vertebral arch screws and osteotomy during surgery increased bleeding and blood pressure was significantly reduced.

Maintenance of blood volume is more important in children as compared with adults, and with equivalent amounts of bleeding, changes in the circulation system in children are more obvious. Therefore, after the patient enters the operating room, balanced salt solution should be actively used to supplement physiologic requirements together with the fluid loss caused by preoperative dietary restrictions [13]. When the patient’s blood volume decreases, auto transfusion should be carried out in a timely manner. If the patient’s circulation fluctuates markedly, a small amount of colloidal solution can be used as a supplement at 5–10 mL/kg [14]. At the same time, transfusion of a red blood cell suspension to improve the hemoglobin concentration, and reduce the influence of hemoglobin loss and hemodilution on the oxygen supply to organs should be carried out according to the patient hemoglobin level. In addition, fresh frozen plasma may be infused according to the patient’s coagulation status [11, 15]. When the hemoglobin concentration is low, the oxygen content is insufficient to meet the oxygen consumption requirements of the brain. At this time, the infusion of concentrated red blood cells can significantly improve the rScO_2_ level in children. Hemoglobin can transport oxygen to various tissues and organs of the body and improve the oxygen carrying capacity of the blood. In scoliosis correction surgery, when the child’s rScO_2_ decreases, in addition to the decrease in cerebral perfusion pressure caused by the decrease in mean arterial pressure, consideration should also be given to whether the child has anemia, a low hemoglobin level, and low oxygen content per unit blood volume. The brain oxygen consumption is greater than the oxygen supply, and the decrease in rScO_2_ caused by the reduced oxygen carrying capacity of hemoglobin may even occur before the blood pressure drops. Therefore, blood gas analysis should be performed in a timely manner, and the appropriate infusion of concentrated red blood cells is essential to improve the blood circulation status in children.

There was no statistically significant relationship in HR and rScO_2_, and this may be related to the physiologic characteristics of children. Due to limited myocardial contractility in children, cardiac output depends mainly on heart rate. When the heart rate slows in children, cardiac output will obviously be reduced, and cerebral perfusion will also decrease. However, in the course of this study, atropine was given when the heart rate decreased due to myocardial depression caused by anesthetic drugs, and slowing of heart rate due to insufficient blood volume was correctable in a timely manner by rapid fluid infusion. As such, patient heart rate was maintained above and below the basic values by about 15%. The effect of surgery on heart rate is transient, and this did not cause a change in cerebral oxygen saturation, and there was therefore no correlation of heart rate with decrease in rScO_2_. It has been reported that the intraoperative PetCO_2_ level is a factor which affects rScO_2_ [16]. A 5–6% increase in rScO_2_ was observed at PetCO_2_ 40–45 mmHg as compared with patient PetCO_2_ of 30–35 mmHg [17]. This is related to the characteristics of cerebral vessels. If the level of CO_2_ is low, the cerebral vessels will contract and cerebral perfusion will be reduced. Conversely, when CO_2_ is high, cerebral perfusion improves and rScO_2_ can be maintained at a high level. In this study, PetCO_2_ remained in a stable state (35–45 mmHg), so there was no correlation in the decrease in rScO_2_ and PetCO_2_ during surgery. Similarly, there was no correlation in SpO_2_ and rScO_2_ reduction in the study period.

There are some limitations in our study. First, this study did not analyze whether there was hypoxic injury in patient organs other than the brain. Second, postoperative follow-up to further analyze whether intraoperative decrease of cerebral oxygen saturation leads to postoperative neurologic complications was not carried out.

## Conclusions

In conclusion, intraoperative cerebral oxygen saturation monitoring can provide effective information to ensure the safety of surgery and anesthesia, and provide a reference for fluid resuscitation. In this study osteotomy for the correction of patients with idiopathic scoliosis resulted in the loss of hemoglobin, decrease in blood pressure and decrease in cerebral oxygen saturation. Under such circumstances, transfusion and fluid infusion should be carried out in a timely manner during surgery, and auto transfusion should be prepared in advance. This will yield improvement in the patient oxygen supply, and maintain a balance between brain oxygen supply and oxygen demand. These are all important steps which may be employed to reduce postoperative complications.

## Data Availability

The datasets used and analysed during the current study are available from the corresponding author on reasonable request.

## Abbreviations

ABP: Arterial blood pressure
AUC: Area under curve
BIS: Bispectral index
BMI: Body mass index
CI: Confidence interval
dHB: Preoperative hemoglobin-postoperative hemoglobin
HR: Heart rate
H-rScO_2_: High regional cerebral oxygen saturation
L-rScO_2_: Low cerebral oxygen saturation groups
NBP: Noninvasive blood pressure
NIRS: Near-infrared spectroscopy
OR: Odds ratio
PetCO_2_: End tidal carbon dioxide partial pressure
ROC: Receiver operating characteristic
rScO_2_: Regional cerebral oxygen saturation
SBP: Systolic blood pressure
SpO_2_: pulse Oxygen saturation
